# Neuroanatomical Features That Predict Response to Electroconvulsive Therapy Combined With Antipsychotics in Schizophrenia: A Magnetic Resonance Imaging Study Using Radiomics Strategy

**DOI:** 10.3389/fpsyt.2020.00456

**Published:** 2020-05-21

**Authors:** Yi-Bin Xi, Long-Biao Cui, Jie Gong, Yu-Fei Fu, Xu-Sha Wu, Fan Guo, Xuejuan Yang, Chen Li, Xing-Rui Wang, Ping Li, Wei Qin, Hong Yin

**Affiliations:** ^1^Department of Radiology, Xijing Hospital, Fourth Military Medical University, Xi'an, China; ^2^Department of Clinical Psychology, School of Medical Psychology, Fourth Military Medical University, Xi'an, China; ^3^School of Life Sciences and Technology, Xidian University, Xi'an, China; ^4^Department of Radiology, Xi'an Mental Health Center, Xi'an, China

**Keywords:** schizophrenia, electroconvulsive therapy, response, prediction, radiomics

## Abstract

**Objective:**

Neuroimaging-based brain signatures may be informative in identifying patients with psychosis who will respond to antipsychotics. However, signatures that inform the electroconvulsive therapy (ECT) health care professional about the response likelihood remain unclear in psychosis with radiomics strategy. This study investigated whether brain structure-based signature in the prediction of ECT response in a sample of schizophrenia patients using radiomics approach.

**Methods:**

This high-resolution structural magnetic resonance imaging study included 57 patients at baseline. After ECT combined with antipsychotics, 28 and 29 patients were classified as responders and non-responders. Features of gray matter were extracted and compared. The logistic regression model/support vector machine (LRM/SVM) analysis was used to explore the predictive performance.

**Results:**

The regularized multivariate LRM accurately discriminated responders from non-responders, with an accuracy of 90.91%. The structural features were further confirmed in the validating data set, resulting in an accuracy of 87.59%. The accuracy of the SVM in the training set was 90.91%, and the accuracy in the validation set was 91.78%.

**Conclusion:**

Our results support the possible use of structural brain feature-based radiomics as a potential tool for predicting ECT response in patients with schizophrenia undergoing antipsychotics, paving the way for utilization of markers in psychosis.

## Introduction

Despite progress, how to treat schizophrenia, an overwhelmingly and highly heterogeneous mental disorder, effectively is still to be learnt because of poor response to antipsychotics in as many as 30% of patients (1). From the perspective of stigmatization, genetic etiology of schizophrenia are more frequently linked to stigmatizing attitudes for psychosis, with a higher level of perceived stigmatization in medical students and medical doctors (2). Effective treatments appear to be critical, thereby lessening the impact of the disorder and decreasing the stigma. Holding synergistic effects in combination with antipsychotics, there is growing evidence to support that addition of electroconvulsive therapy (ECT) may be an effective alternative for chronic patients with refractory symptoms, exhibiting the safety and short-term efficacy (3–5). ECT is most commonly considered in patients with schizophrenia only after unsuccessful treatment with antipsychotic medication (6). Also, ECT may be of value in the treatment of acute schizophrenia when used in combination with antipsychotic medications (7).

Previous clinical studies also demonstrate that a small portion of these patients are resistant to substantial improvement with ECT of clozapine augmentation. Overall, 34% non-responder rate has been reported by a meta-analysis recently; 46% non-responder rate across clinical trials (8). Because ECT is liable to provoking cognitive impairment and multi-system adverse effects, much more invasive relative to pharmacotherapy, costly in relation to the procedure, and available for partial hospitals, the ECT health care professional should accurately identify non-responders before treatment. A pressing need of predictive markers exists in clinical management of ECT for schizophrenia, which is of high clinical relevance. A recent review has shown the predictors of response to ECT in schizophrenia, including symptoms, age, duration of illness, family history of schizophrenia, and baseline global functioning and cognitive functions (9), but no further attempts have been made to substantiate the predictors.

Neuroimaging techniques provide promising assessment tools to allow for individualized treatment in psychiatry. However, despite compelling evidence of a biological basis of schizophrenia, research into brain imaging features reflecting its pathophysiology mechanism to guide clinical practice is obviously lagged behind (10). Substantial efforts are still required to identify biomarkers informative in treatment response before reaping the reward of clinical benefit in schizophrenia patients. Decades-long research on structural and functional imaging has accumulated burgeoning evidence for disrupted brain network involving the pathophysiology of schizophrenia. In a previous review by Dazzan et al. (11), structural magnetic resonance imaging (MRI) could be helpful in stratifying patients with schizophrenia into clinically meaningful clusters. Afterward, despite negative results (12), proliferated evidence has provided more support for structural features of responsiveness to antipsychotic drug treatment (see Cui et al. for review) (13).

Promisingly, according to several recent findings, neuroimaging could be helpful identifying the predictive biological markers of depression. Most neuroimaging studies of ECT demonstrate treatment-related increase of hippocampal volume (14) and disturbed white matter integrity (15). In major depressive disorder, Oltedal et al. detected 0.28% hippocampal enlargement per ECT session, and the variety of volume change by electrode placement (i.e., bilateral, right unilateral) in the left hippocampus; Repple et al. found an increased mean diffusivity following ECT in the right hemisphere, and a correlation between seizure duration and decreased fractional anisotropy, suggesting an effect of ECT on the permeability of the blood-brain barrier. A recent structural MRI at baseline has been proven to be successful for predicting responsiveness to ECT in patients with major depressive disorder (16). As for functional MRI, in severe and treatment-resistant depressive patients, a multivariate pattern analysis study suggests that resting-state networks could play an important role in predicting remission from depression following a course of ECT (17). A review by Sanghani et al. summarizes neuroimaging predictors of response to ECT augmentation of antipsychotic medications in schizophrenia, involving structural and functional MRI (4). Also, radiomics approach could play an important role in precision medicine (https://allofus.nih.gov/). In the context of radiomics, it is a method to obtain more high dimensional features that might be options used for machine learning analysis, because medical images are digitally encrypted and hold a number of information involved in pathophysiology (18). It extracts high-throughput information and detects core features for supporting clinical decision making. Radiomics analysis has shown evident capacities for classification and prediction in schizophrenia. Notably, a valid approach by means of MRI to diagnose schizophrenia has been developed using radiomics strategy with an accuracy of 87%, as demonstrated by Cui et al. (19). Taken together, neuroimaging-based brain signatures hold great promise that contributes to supporting decision making to select the proper treatment in psychosis. The structural underpinnings are crucial for underling the pathophysiology of schizophrenia (20–23). It is already plausible that cerebral structure-based neurologic signature could be a potential biomarker associated with treatment response for patients with schizophrenia.

Therefore, in the current study, we aimed to examine whether brain structure-based signature in the prediction of ECT response in a sample of schizophrenia patients using radiomics approach, making a step to improve individualized treatment of schizophrenia using specific, quantitative, and objective biomarker for clinical management. Both logistic regression model and support vector machine (LRM/SVM) analyses were used to explore the predictive performance. In line with Tandon et al. (24), our goal is also “a deeper understanding of the pathophysiology of schizophrenia leading to improved treatment”. We hypothesized patients with a distinct structural feature of the brain would reveal different response to ECT.

## Material and Methods

### Participants

This study was approved by the local institutional ethics committee. Written informed consent was obtained from all the participants (or their parents for those under age of 18 years) after complete description of the study. A total of 57 patients who would be assigned to take ECT were recruited from the Xi'an Mental Health Center (25). According to clinical practice guidelines and expert consensus, indications for use of ECT include patients with treatment-resistant schizophrenia or with schizophrenia who have suicidal behavior/suicide attempts/acute episode (see the **Supplementary Material**). Treatment resistance is defined as little or no symptomatic response to multiple (at least two) antipsychotic trials of an adequate duration (at least 6 weeks) and dose (therapeutic range) [American Psychiatric Association (APA) practice guidelines] (26). The consensus diagnoses were made by two experienced clinical psychiatrists on the basis of Structured Clinical Interview for Diagnostic and Statistical Manual of Mental Disorders, Fourth Edition, Text Revision (DSM-IV-TR) using all the available information. All patients underwent MRI scanning at baseline. Our study had no influence on the therapy and clinical assessment determined by their clinicians. Positive And Negative Syndrome Scale (PANSS) that evaluated each patient's symptoms at the time of first and second scans were used for the following analysis. The exclusion criteria included: (1) presence of another axis I or axis II psychiatric disorder; (2) history of receiving ECT; (3) history of clinically significant neurological, neurosurgical, or medical illnesses; (4) substance abuse within the prior 30 d or substance dependence within the prior 6 months; (5) pregnancy or other MR imaging contraindications, e.g., cardiac pacemakers and other metallic implants.

Treatment response was assessed using percentage change of symptoms based on PANSS. Responders were defined as 70% reduction in PANSS total scores, an elevated level as high as the criterion of 30% traditionally used (27, 28), as performed by Petrides et al. (29). In our database, only subscale scores were available, we were unable to use the Remission criteria of the Schizophrenia Working Group Consensus (30). Here, we must emphasize the additional complexity and risk associated with ECT, as well as mounted efforts from clinicians and patients, and therefore we increased expectations with which should be met. Dose of antipsychotic medication was converted to defined daily dose (DDD) (31). Patients were divided into two groups randomly, a training set (n = 44, including 22 responders and 22 non-responders) and a validation set (n = 13, including 6 responders and 7 non-responders) using statistical software. Demographic and clinical characteristics are listed in **Table 1**.

**Table 1:** Demographical and clinical characteristics of participants.

Characteristic	Responders (n = 28)	Non-Responders (n = 29)	*P* values
Age (y)	31.0 ± 10.2	29.7 ± 8.5	.61
Gender (M/F)	17/11	20/9	.51
Education level (y)	11.7 ± 3.5	11.3 ± 4.0	.71
Duration of illness (y)	5.4 ± 6.4	6.1 ± 6.4	.67
Time between measurements (w)^a^	3.9 ± 1.1	3.9 ± 1.1	.89
CGI score at baseline	5.6 ± 1.1	5.7 ± 0.6	.72
CGI score after ECT	2.6 ± 0.7	3.6 ± 0.9	<.001
PANSS score at baseline			
Positive score	29.9 ± 6.2	28.0 ± 8.1	.32
Negative score	19.5 ± 10.3	29.0 ± 10.9	.001
General score	44.7 ± 12.1	41.7 ± 9.5	.31
Total score	94.1 ± 19.2	98.7 ± 21.0	.39
PANSS score after ECT			
Positive score	9.7 ± 2.0	15.4 ± 3.5	<.001
Negative score	10.6 ± 4.9	19.7 ± 7.9	<.001
General score	19.7 ± 3.4	28.6 ± 6.7	<.001
Total score	40.0 ± 7.1	63.7 ± 13.8	<.001
Changes in PANSS score	84.7% ± 9.6%	51.0% ± 12.8%	<.001
Number of ECT^a^	10.3 ± 2.0	10.0 ± 2.9	.67
Antipsychotic dose (mg/d)^b^	17.5 ± 5.9	13.8 ± 6.7	.03

a Data missing for one non-responder.

b Olanzapine equivalents based on defined daily doses method.

CGI, Clinical Global Impressions; ECT, electroconvulsive therapy; PANSS, Positive And Negative Syndrome Scale.

### Image Acquisition

A GE Discovery MR750 3.0 T scanner was used to acquire images in the Department of Radiology at Xi'an Mental Health Center. As we performed previously, high-resolution T1-weighted structural data were obtained as we performed previously (**Table S1**) (19). During the scans, all participants were instructed to relax, move and think of nothing in particular as little as possible, keep their eyes closed, and not sleep. We also used a custom-built head cushion to minimize head motion in order to avoid excessive motion, reducing head motion artifacts during acquisition. Thereafter, we carefully checked all the images before the following analysis, thereby ensuring the image quality. Further details about image acquisition are described in the **Supplementary Material**.

### Electroconvulsive Therapy

Unilateral ECT was conducted in all patients three cycles per week within the duration of hospital stay. A total of nine to 12 sessions of ECT were given using an instrument (spECTrum 5000Q). The stimulus current was 800 mA, and the stimulus duration ranged from 3 to 5 s. Stimulus frequency was adapted due to different seizure threshold varied for individuals. Also, electrocardiography, blood pressure, and oxygen saturation examinations were carried out to exclude severe complications. All patients were anesthetized with propofol or etomidate, and a muscle relaxant (succinylcholine) was administered, which were calculated based on the body weight of each patient.

### Image Processing

**Figure 1:**
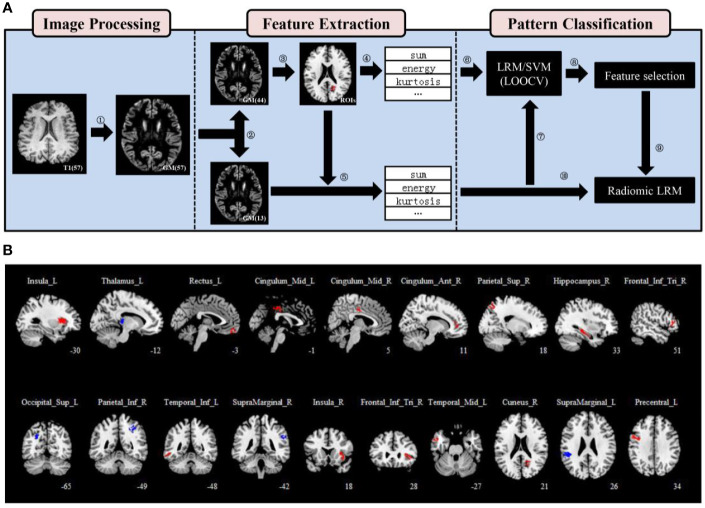
Regions of interest (ROIs) definition and feature extraction. **(A)** A flowchart for the data processing. (1) The modulated and normalized gray matter (GM) tissues were generated from T1-weighted images of each patient. (2) Patients were divided into two groups randomly, a training set (n = 44) and a validation set (n = 13) using statistical software. (3) Nineteen ROIs were defined using a two-sample *t*-test and two thresholds. (4), (5) 15 first-order statistics features were extracted from each ROI of patients in both the training and validation sets. (6), (7) A leave-one-out cross-validation (LOOCV) framework was used to perform pattern classification analysis in the training set, and all models were validated on the validation set. (8) We calculated the frequency of each feature and obtained a ranking that characterized the importance of features. (9), (10) three selected features were used to train the radiomic LRM. **(B)** Nineteen ROIs were defined using a two-sample *t*-test, a threshold of *P* < .05 (uncorrected) and an extent threshold of 100 voxels. The anatomical location of each ROI was described using AAL atlas.

Data processing is described elsewhere previously (**Figure 1A**). The voxel-based morphometry (VBM) analysis was performed using CAT12 toolbox (C. Gaser, Structural Brain Mapping group, Jena University Hospital, Jena, Germany) implemented in SPM12 (Statistical Parametric Mapping, Institute of Neurology, London, UK) with the default setting. Briefly, All T1-weighted images are normalized using an affine followed by non-linear registration, corrected for bias field in homogeneities. Segmentation into gray matter (GM), white matter (WM), and cerebrospinal fluid (CSF) (32) and spatially normalization using the DARTEL-algorithm (33) were then applied. In the last step of DARTEL, a non-linear deformation approach was used to modulate the GM tissues for comparing the relative GM volume adjusted for individual brain size. Furthermore, the voxel values in the tissue maps are modulated by the Jacobian determinant that was calculated during spatial normalization (34). To assess the homogeneity of the GM tissues, a quality check was performed using a CAT12 toolbox once the preprocessing pipeline was completed (35). The modulated and normalized GM tissues were not smoothed to avoid loss of image information. The GM tissues were used for subsequent statistical analysis.

### Statistical Analysis and ROIs Definition

We used SPM12 for all of the statistical analyses. First, a two-sample *t*-test was performed to compare the GM volume between responder and non-responder in the training set without covariates. The relative GM volume changes were assessed at a threshold of *P* < .05 uncorrected. The extent threshold was set at 100 voxels to define the regions of interest (ROIs) which could have enough voxels to calculate features for classification. Focusing on the ROIs from the two-sample *t*-test is equivalent to initially screening out areas that may be distinguishable, so that their features may have clinically meaningful predictive capacity. The Automated Anatomical Labeling (AAL) atlas (36), which parceled the GM into 90 anatomical regions was adopted to describe the anatomical location of each ROI. If the overlapping percentage of the ROI with a region of AAL is greater than 50%, the anatomical location of it is described as the region (**Table S2**).

### Feature Extraction

Fifteen first-order statistics features calculated from the histogram of each ROI GM volume values were extracted from each ROI of patients in both the training and validation sets (**Table S3**) (37). The mean, median, standard deviation, and root mean square are the most commonly used and basic statistical metrics. The skewness measures the degree of histogram asymmetry around the mean, and kurtosis is a measure of the histogram sharpness. As measures of histogram randomness we computed the uniformity and entropy of the image histogram. The feature algorithms were implemented in Matlab 2014a (MathWorks, Natick, MA, USA). All features of ROIs for each patient were concatenated into a feature vector and were connected in parallel to form a feature matrix in each data set. All features of the training set were standardized to zero mean and unit variance to avoid model building being affected by the differences in the feature scales, and the validation set were processed with the same standardization criterion to ensure the independence before pattern classification (38).

### Pattern Classification Analysis

**Figure 2:**
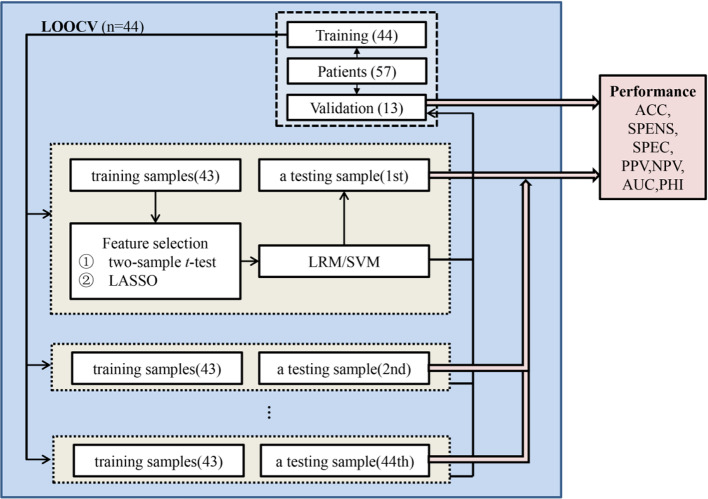
Pattern classification analysis. A leave-one-out cross-validation (LOOCV) framework was used to perform pattern classification analysis in the training set. In the LOOCV, one patient was used as a testing sample, and the remaining patients were applied as training samples to select features and build the classifier to classify the testing sample. Classification performance could be estimated based on all of the testing samples and be validated based on the averaged classification results of validation set. ACC, accuracy; SENS, sensitivity; SPEC, specificity; PPV, positive predictive value; NPV, negative predictive value; AUC, area under the receiver operating characteristic curve; PHI, phi correlation coefficient.

A leave-one-out cross-validation (LOOCV) framework was used to perform pattern classification analysis in the training set (**Figure 2**) (39). Specifically, the best features based on univariate statistical tests (two-sample *t*-test) between responders and non-responders in the remaining patients were selected with *P* < .05 (uncorrected), and then regularized multivariate logistic regression model (LRM) with the least absolute shrinkage and selection operator (LASSO) penalty was applied to select more important features and to build a classifier (40). Moreover, the support vector machine (SVM) method was used to validate results as an independent classifier using the features selected by LASSO. Performance of the classifiers was measured quantitatively using the area under the receiver operating characteristic (ROC) curve (AUC) (**Table S4**). Besides, one patient did not receive antipsychotic drugs, which might affect the results. Since there had been a significant difference in antipsychotic dose between responders and non-responders, we excluded this patient and reanalyzed.

In order to obtain a model with fixed radiomic features and analyze the impact of clinical factors, we constructed the multivariate LRM based on the training set and validated it on the validation set. Frequency of each feature selected by LASSO across 44 training partitions of the cross-validation setup was calculated to assess feature importance and to obtain a ranking of features (**Table S5**) (41). Features greater than 50% of the selected frequency were important and used as a feature set for further feature selection (41). To analyze the impact of clinical factors, negative score and antipsychotics dose combined with fixed radiomic features were also used to build the fusion LRM model. For these two LRM, we evaluated the multicollinearity according to the variance inflation factor (VIF) and tested the significance of regression coefficients (β) with *t*-tests for each independent variable (42). If VIF was less than 4, there was no evidence of a multicollinearity problem (42). The following R packages were used for pattern classification analysis (http://www.R-project.org) (see **Supplementary Material** for this section).

## Results

### Participant Characteristics

The demographic and clinical characteristics of the participants are listed in **Table 1**. In consistence with previous reports (13), 28 and 29 patients were classified as responders (49%) and non-responders (51%) after ECT. No statistically significant difference in baseline demographic characteristics was found between responders and non-responders. Participants who responded had a lower level of PANSS negative score (*P* = .001) and received more antipsychotics (*P* = .03) than those who did not respond before treatment.

### ROIs Definition and Feature Extraction

Nineteen ROIs were defined, and the details were described in **Table S2** and were shown in **Figure 1B**. Fifteen first-order statistics features were calculated from the histogram of each ROI GM volume values (**Table S3**). A total of 285 features per patient were used for pattern classification analysis.

### Predictive Performance

**Figure 3:**
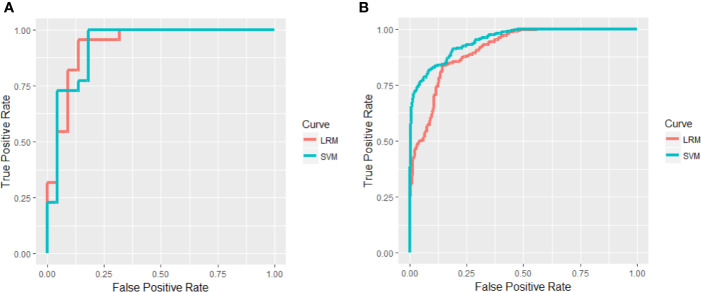
Areas under receiver operating characteristic (ROC) curves in the training set **(A)** and validation set **(B)**.

The regularized multivariate LRM accurately discriminated responders from non-responders on the basis of the ROC curve with an accuracy of 90.91% and AUC value of 0.9318 (**Figure 3A** and **Table S4**) and were further confirmed in the validating data set, resulting in an accuracy of 87.59% and AUC value of 0.9031 (**Figure 3B** and **Table S4**). To assess possible overfitting, the DeLong test was implemented on the ROC curves and revealed that the differences were not statistically significant among the AUCs of the training set and the validation set, with *P* values of 0.5804. Moreover, we used the SVM to replicate findings above. The accuracy of the SVM based on all of the testing samples in the training set was 90.91%, and the accuracy in the validation set was 91.78% (**Table S4**). The ROC curves of the training set and the validation set were shown in **Figures 3A, B**, and *P* value of the DeLong test was 0.6443. Moreover, we excluded a patient without receiving antipsychotics and reanalyzed, and there was no significant difference in antipsychotic dose between responders and non-responders (*P* > 0.05). The accuracy of the LRM based on all of the testing samples in the training set was 90.91%, and the accuracy in the validation set was 87.12% (**Table S6**). The AUC of the training set and the validation set were 0.9318 and 0.9034 separately, and *P* value of the DeLong test was 0.5586. The accuracy of the SVM based on all of the testing samples in the training set was 90.91%, and the accuracy in the validation set was 90.91% (**Table S6**). The AUC of the training set and the validation set were 0.9298 and 0.9407 separately, and *P* value of the DeLong test was 0.8386.

The fusion LRM with fixed radiomic features was built (**Table S7**) and the classification performance of the model was shown in **Table S8**. The ROC curves were shown in **Figure S1** and *P* value of the DeLong test was 0.3785. Negative score and antipsychotics dose combined with fixed radiomic features were also used to build the fusion LRM model for analyzing the impact of clinical factors. However, negative score was removed due to the multicollinearity (VIF > 4), and antipsychotics dose was removed because the regression coefficient was not significant. These two clinical factors could not be left in the final LRM.

## Discussion

In this naturalistic study, the principal findings indicated a potential predictive capacity between patients who subsequently responded and did not respond using radiomics approach, with clinically meaningful accuracy (LRM: 90.91% in training and 87.59% in validation; SVM: 90.91% in training and 91.78% in validation). We identified neuroanatomical features with successful level prediction of ECT response, involving cortical (inferior frontal gyrus, cingulate cortex, and temporal and parietal lobes) and subcortical regions (insula, thalamus, and hippocampus).

Although ECT combined with antipsychotics has been included in treatment guidelines (26, 43), the suggestions from radiologists to psychiatrists and patients and their families regarding the treatment efficacy of ECT based on predictive/prognostic markers, as approaches uncovering paths toward delivering precision medicine, is of remarkable clinical significance. In the study by Kupchik et al., there are 33% (n = 12) patients with symptoms resistant to typical antipsychotics who did not benefit in the clozapine-ECT group (44). Then, an open-label prospective study finds that 40% (n = 6) patients with refractory schizophrenia did not meet response criteria after receiving a combination of ECT and clozapine. Furthermore, a prospective randomized study notes 51.3% (n = 20) patients defined as non-responders after treatment of ECT plus clozapine or ECT augmentation (29). In a meta-analysis reporting 46% non-responder rate across clinical trials, the response defined as percentage improvement in scale/subscale score of PANSS/Brief Psychiatric Rating Scale, arranging from 25% to 40% (8). On the contrary, we defined as decrease of ≥ 70% in total PANSS score, resulting in higher level of non-responder rate (51%). Radiomics features in the present study may assist in the treatment selection, allowing for individual differences in treatment algorithms to some extent. Hence, additional remedies need to be considered and much more attention need to be paid in such a setting to ameliorate symptoms of patients, including transcranial magnetic stimulation, and psychological therapy. Biomarkers obtained from the brain such as structural features hold great promise that contributes to better management of patients with refractory schizophrenia.

Of particular interest, in our study, features used for prediction were extracted predominantly from the inferior frontal gyrus, cingulate cortex, temporal and parietal lobes, insula, thalamus, and hippocampus. These identified regions have been implicated in previous studies looking before/after ECT brain changes. Recent MRI studies have detected longitudinal alterations of the brain pre- and post-ECT interventions in patients with schizophrenia. Recent neuroimaging studies have shown an association of ECT in schizophrenia with increase in GM volume within the medial temporal lobe (amygdala, hippocampus, and insula) (45) and increased global functional connectivity density among key regions within default mode network (precuneus and medial prefrontal cortex) (46). ECT exerts therapeutic efficacy *via* elevating the concentration of *N*-acetylaspartate in the left prefrontal cortex and thalamus, indicating an antipsychotic effects through neuroprotective mechanism (47). In a sample of schizophrenia and depression, ECT reveals both disorder-specific and transdiagnostic effects. Structural network analysis suggests a volume increase in the lateral prefrontal/cingulate cortical network as schizophrenia-specific changes after ECT (48). On the contrary, transdiagnostic impacts across schizophrenia and depression include an enlargement of the medial temporal lobe (hippocampus, parahippocampus, and amygdala) and insula, together with attenuated functional connectivity involving tempoparietal, prefrontal, and cortical midline structures and augmented hypothalamic functional connectivity (45, 48). It may be why these regions (therapeutic target) predict treatment outcome.

Methodologically, both LRM and SVM are linear classifiers. Using both of them demonstrates the validity of results. Machine-learning classification studies will have an influence on precision medicine. The increasing importance of radiomics in medical imaging creates an ideal situation for application of radiomics in psychiatry where there is no “lesion” but there are “features” for mental disorders, i.e., brain structure derived from structural MRI. This step by step analysis could promote our findings transforming clinical practice, which is helpful for the generalizability of the potential marker. Most recently, classification approaches provide a powerful way to promote the translation of neuroimaging-based signature to diagnostic, predictive or prognostic biological markers for mental disorders, including schizophrenia (19). In addition to these structural and functional tests previously discovered, we proposed this novel predictive tool to replenish the existing clusters of potential biomarkers, as an alternative approach. Future research needs to integrate and optimize them, resulting in true cerebral markers for clinical management of schizophrenia.

Nevertheless, there are still several issues that merit comments. The clinical characteristics revealed a variable duration of illness and antipsychotic dose for schizophrenia patients, which might have impact on our results. As a routine and natural clinical phenomenon, we could not modulate patients' duration of illness. In our study, we randomly divided patients in a 3:1 ratio to obtain a training set of 44 patients and a validation set of 13 patients. In order to overcome the issue of sample size and provide reliable prediction results, we used the LOOCV method in the training set to evaluate the classification ability of GM features, and select important features to construct a final model. Likewise, previous machine learning studies used similar sample sizes (25 responders and 13 non-responders in first-episode drug-naive patients with schizophrenia; 13 responders and 10 non-responders in patients with acute major depressive disorder) and got meaningful results (accuracy of 78.6% and 78.3%/73.9%) (16, 49). The number of features is much larger than the sample size, and the training set and validation set are drawn from the same dataset. Although we use the LASSO for feature selection to reduce the number of features and divide the data sets randomly, single-center data may lead to an overly optimistic estimation of classification performance and limit the generalizability of the results. In the future, multi-center datasets with larger samples will be needed to validate and promote these findings. Furthermore, we were unable to analyze the life-time cumulative dosages of antipsychotics that were unavailable in the clinical database at the hospital. The difference is small in the effectiveness of individual antipsychotics (50), and responders and non-responders in this study received antipsychotics in doses recommended by APA Practice Guidelines (olanzapine equivalents, 17.5 ± 5.9 mg/d, 13.8 ± 6.7 mg/d), which proposes that olanzapine in doses of 10–20 mg/d is effective in the acute phase of schizophrenia (26). Despite majority of atypical antipsychotics, patients received heterogeneous drugs, which may be helpful to control for drug dose or treatment type (e.g., typical antipsychotic and atypical antipsychotic) in the future analysis. Additionally, there was a significant difference in the severity of negative symptoms between responders and non-responders before treatment. This difference begs the question: would the baseline PANSS negative score be a predictor of treatment response? But **Figure S2** indicates a limited diagnostic performance.

## Conclusion

Conclusively, this study implies that radiomics-based structural brain feature could predict response to ECT combined with antipsychotics in schizophrenia patients. Useful neuroanatomical features for prediction involve brain regions modulated by ECT. Additional effort is urgent for longitudinal differences after the ECT series in patients with schizophrenia.

## Data Availability Statement

The datasets for this article are not publicly available because no agreement with regard to public availability was received from the participants in this study. Requests to access the datasets should be directed to L-BC, lbcui@fmmu.edu.cn.

## Ethics Statement

The studies involving human participants were reviewed and approved by institutional ethics committee at Xi'an Mental Health Center. Written informed consent to participate in this study was provided by the participants' legal guardian/next of kin.

## Author Contributions

L-BC, HY, and WQ conceptualized the study. Y-BX, L-BC, and JG wrote the first draft of manuscript and conducted the statistical analyses. Y-FF, X-SW, FG, XY, CL, X-RW, and PL collected and organized the primary data. Y-BX, HY, and WQ designed the study and provided supervision in the implementation of the study. All authors have approved the final manuscript.

## Funding

The author(s) disclosed receipt of the following financial support for the research, authorship and/or publication of this article: This work was supported by grants 81801675 from the National Natural Science Foundation of China and 2019CYJH from the Fourth Military Medical University (L-BC), 81571651 from the National Natural Science Foundation of China and 2017ZDXM-SF-048 from Key Research and Development Program of Shaanxi Province (HY), grants 2015CB856403 and 2014CB543203 from the National Basic Research Program of China, grant 201809170CX11JC12 from the Science and Technology Projects of Xi'an, and grants 81771918, 81471811, and 81471738 the National Natural Science Foundation of China (WQ).

## Conflict of Interest

The authors declare that the research was conducted in the absence of any commercial or financial relationships that could be construed as a potential conflict of interest.

## References

[1] Freedman R Schizophrenia. N Engl J Med (2003) 349:1738–49. 10.1056/NEJMra035458 14585943

[2] Serafini G Pompili M Haghighat R Pucci D Pastina M Lester D Stigmatization of schizophrenia as perceived by nurses, medical doctors, medical students and patients. J Psychiatr Ment Health Nurs (2011) 18:576–85. 10.1111/j.1365-2850.2011.01706.x 21848591

[3] Weiner RD Reti IM Key updates in the clinical application of electroconvulsive therapy. Int Rev Psychiatry (2017) 29:54–62. 10.1080/09540261.2017.1309362 28406327

[4] Sanghani SN Petrides G Kellner CH Electroconvulsive therapy (ECT) in schizophrenia: a review of recent literature. Curr Opin Psychiatry (2018) 31:213–22. 10.1097/YCO.0000000000000418 29528902

[5] Ahmed S Khan AM Mekala HM Venigalla H Ahmed R Etman A Combined use of electroconvulsive therapy and antipsychotics (both clozapine and non-clozapine) in treatment resistant schizophrenia: A comparative meta-analysis. Heliyon (2017) 3:e00429. 10.1016/j.heliyon.2017.e00429 29264404 PMC5727374

[6] APA The Practice of Electroconvulsive Therapy: Recommendations for Treatment, Training, and Privileging: A Task Force Report of the American Psychiatric Association. 2nd ed Washington, DC: American Psychiatric Association (2002). 10.1097/PRA.0000000000000914 41894194

[7] Galletly C Castle D Dark F Humberstone V Jablensky A Killackey E Royal Australian and New Zealand College of Psychiatrists clinical practice guidelines for the management of schizophrenia and related disorders. Aust N Z J Psychiatry (2016) 50:410–72. 10.1177/0004867416641195 27106681

[8] Lally J Tully J Robertson D Stubbs B Gaughran F MacCabe JH Augmentation of clozapine with electroconvulsive therapy in treatment resistant schizophrenia: A systematic review and meta-analysis. Schizophr Res (2016) 171:215–24. 10.1016/j.schres.2016.01.024 26827129

[9] Grover S Sahoo S Rabha A Koirala R ECT in schizophrenia: a review of the evidence. Acta Neuropsychiatr (2019) 31:115–27. 10.1017/neu.2018.32 30501675

[10] Keefe RSE Kahn RS Cognitive Decline and Disrupted Cognitive Trajectory in Schizophrenia. JAMA Psychiatry (2017) 74:535–6. 10.1001/jamapsychiatry.2017.0312 28329400

[11] Dazzan P Arango C Fleischacker W Galderisi S Glenthoj B Leucht S Magnetic resonance imaging and the prediction of outcome in first-episode schizophrenia: a review of current evidence and directions for future research. Schizophr Bull (2015) 41:574–83. 10.1093/schbul/sbv024 PMC4393706 25800248

[12] Doucet GE Moser DA Luber MJ Leibu E Frangou S Baseline brain structural and functional predictors of clinical outcome in the early course of schizophrenia. Mol Psychiatry (2020) 25:863–72. 10.1038/s41380-018-0269-0 PMC6447492 30283030

[13] Cui LB Cai M Wang XR Zhu YQ Wang LX Xi YB Prediction of early response to overall treatment for schizophrenia: A functional magnetic resonance imaging study. Brain Behav (2019) 9:e01211. 10.1002/brb3.1211 30701701 PMC6379641

[14] Oltedal L Narr KL Abbott C Anand A Argyelan M Bartsch H Volume of the Human Hippocampus and Clinical Response Following Electroconvulsive Therapy. Biol Psychiatry (2018) 84:574–81. 10.1016/j.biopsych.2018.05.017 PMC6697556 30006199

[15] Repple J Meinert S Bollettini I Grotegerd D Redlich R Zaremba D Influence of electroconvulsive therapy on white matter structure in a diffusion tensor imaging study. Psychol Med (2020) 50:849–56. 10.1017/S0033291719000758 31010441

[16] Redlich R Opel N Grotegerd D Dohm K Zaremba D Burger C Prediction of Individual Response to Electroconvulsive Therapy via Machine Learning on Structural Magnetic Resonance Imaging Data. JAMA Psychiatry (2016) 73:557–64. 10.1001/jamapsychiatry.2016.0316 27145449

[17] van Waarde JA Scholte HS van Oudheusden LJ Verwey B Denys D van Wingen GA A functional MRI marker may predict the outcome of electroconvulsive therapy in severe and treatment-resistant depression. Mol Psychiatry (2015) 20:609–14. 10.1038/mp.2014.78 25092248

[18] Lambin P Leijenaar RTH Deist TM Peerlings J de Jong EEC van Timmeren J Radiomics: the bridge between medical imaging and personalized medicine. Nat Rev Clin Oncol (2017) 14:749–62. 10.1038/nrclinonc.2017.141 28975929

[19] Cui LB Liu L Wang HN Wang LX Guo F Xi YB Disease Definition for Schizophrenia by Functional Connectivity Using Radiomics Strategy. Schizophr Bull (2018) 44:1053–9. 10.1093/schbul/sby007 PMC6101635 29471434

[20] Dietsche B Kircher T Falkenberg I Structural brain changes in schizophrenia at different stages of the illness: A selective review of longitudinal magnetic resonance imaging studies. Aust N Z J Psychiatry (2017) 51:500–8. 10.1177/0004867417699473 28415873

[21] Brugger SP Howes OD Heterogeneity and Homogeneity of Regional Brain Structure in Schizophrenia: A Meta-analysis. JAMA Psychiatry (2017) 74:1104–11. 10.1001/jamapsychiatry.2017.2663 PMC5669456 28973084

[22] Palaniyappan L Progressive cortical reorganisation: A framework for investigating structural changes in schizophrenia. Neurosci Biobehav Rev (2017) 79:1–13. 10.1016/j.neubiorev.2017.04.028 28501553

[23] Liu S Li A Liu Y Yan H Wang M Sun Y Polygenic effects of schizophrenia on hippocampal grey matter volume and hippocampus-medial prefrontal cortex functional connectivity. Br J Psychiatry (2020) 216:267–74. 10.1192/bjp.2019.127 31169117

[24] Tandon N Tandon R Will Machine Learning Enable Us to Finally Cut the Gordian Knot of Schizophrenia. Schizophr Bull (2018) 44:939–41. 10.1093/schbul/sby101 PMC6101563 29986110

[25] Gong J Cui LB Xi YB Zhao YS Yang XJ Xu ZL Predicting response to electroconvulsive therapy combined with antipsychotics in schizophrenia using multi-parametric magnetic resonance imaging. Schizophr Res (2019) S0920-9964(19):30553–5. 10.1016/j.schres.2019.11.046 (In press) 31826827

[26] Lehman AF Lieberman JA Dixon LB McGlashan TH Miller AL Perkins DO Practice guideline for the treatment of patients with schizophrenia, second edition. Am J Psychiatry (2004) 161:1–56 10.1016/S0277-9536(03)00261-2 15000267

[27] Leucht S Arbter D Engel RR Kissling W Davis JM How effective are second-generation antipsychotic drugs? A meta-analysis of placebo-controlled trials. Mol Psychiatry (2009) 14:429–47. 10.1038/sj.mp.4002136 18180760

[28] Beitinger R Lin J Kissling W Leucht S Comparative remission rates of schizophrenic patients using various remission criteria. Prog Neuropsychopharmacol Biol Psychiatry (2008) 32:1643–51. 10.1016/j.pnpbp.2008.06.008 18616969

[29] Petrides G Malur C Braga RJ Bailine SH Schooler NR Malhotra AK Electroconvulsive therapy augmentation in clozapine-resistant schizophrenia: a prospective, randomized study. Am J Psychiatry (2015) 172:52–8. 10.1176/appi.ajp.2014.13060787 25157964

[30] Andreasen NC Carpenter WT Jr. Kane JM Lasser RA Marder SR Weinberger DR Remission in schizophrenia: proposed criteria and rationale for consensus. Am J Psychiatry (2005) 162:441–9. 10.1176/appi.ajp.162.3.441 15741458

[31] Leucht S Samara M Heres S Davis JM Dose Equivalents for Antipsychotic Drugs: The DDD Method. Schizophr Bull (2016) 42(Suppl 1):S90–4. 10.1093/schbul/sbv167 PMC4960429 27460622

[32] Ashburner J Friston KJ Unified segmentation. Neuroimage (2005) 26:839–51. 10.1016/j.neuroimage.2005.02.018 15955494

[33] Ashburner J A fast diffeomorphic image registration algorithm. Neuroimage (2007) 38:95–113. 10.1016/j.neuroimage.2007.07.007 17761438

[34] Good CD Johnsrude IS Ashburner J Henson RN Friston KJ Frackowiak RS. A voxel-based morphometric study of ageing in 465 normal adult human brains. Neuroimage (2001) 14:21–36. 10.1006/nimg.2001.0786 11525331

[35] Coppola G Petolicchio B Di Renzo A Tinelli E Di Lorenzo C Parisi V Cerebral gray matter volume in patients with chronic migraine: correlations with clinical features. J Headache Pain (2017) 18:115. 10.1186/s10194-017-0825-z 29322264 PMC5762618

[36] Tzourio-Mazoyer N Landeau B Papathanassiou D Crivello F Etard O Delcroix N Automated anatomical labeling of activations in SPM using a macroscopic anatomical parcellation of the MNI MRI single-subject brain. Neuroimage (2002) 15:273–89. 10.1006/nimg.2001.0978 11771995

[37] Aerts HJ Velazquez ER Leijenaar RT Parmar C Grossmann P Carvalho S Decoding tumour phenotype by noninvasive imaging using a quantitative radiomics approach. Nat Commun (2014) 5:4006. 10.1038/ncomms5644 24892406 PMC4059926

[38] Kuhn M Johnson K Data pre-processing. Applied predictive modeling. 1st ed New York: Springer (2013) p. 27–59.

[39] Fan Y Liu Y Wu H Hao Y Liu H Liu Z Discriminant analysis of functional connectivity patterns on Grassmann manifold. Neuroimage (2011) 56:2058–67. 10.1016/j.neuroimage.2011.03.051 21440643

[40] Zhang B Tian J Dong D Gu D Dong Y Zhang L Radiomics Features of Multiparametric MRI as Novel Prognostic Factors in Advanced Nasopharyngeal Carcinoma. Clin Cancer Res (2017) 23:4259–69. 10.1158/1078-0432.CCR-16-2910 28280088

[41] Dwyer DB Cabral C Kambeitz-Ilankovic L Sanfelici R Kambeitz J Calhoun V Brain Subtyping Enhances The Neuroanatomical Discrimination of Schizophrenia. Schizophr Bull (2018) 44:1060–9. 10.1093/schbul/sby008 PMC6101481 29529270

[42] Kabacoff RI R in Action: Data Analysis and Graphics with R Second Edition. Manning Publications (2015).

[43] Hasan A Falkai P Wobrock T Lieberman J Glenthoj B Gattaz WF World Federation of Societies of Biological Psychiatry (WFSBP) Guidelines for Biological Treatment of Schizophrenia, part 1: update 2012 on the acute treatment of schizophrenia and the management of treatment resistance. World J Biol Psychiatry (2012) 13:318–78. 10.3109/15622975.2012.696143 22834451

[44] Kupchik M Spivak B Mester R Reznik I Gonen N Weizman A Combined electroconvulsive-clozapine therapy. Clin Neuropharmacol (2000) 23:14–6. 10.1097/00002826-200001000-00003 10682225

[45] Thomann PA Wolf RC Nolte HM Hirjak D Hofer S Seidl U Neuromodulation in response to electroconvulsive therapy in schizophrenia and major depression. Brain Stimul (2017) 10:637–44. 10.1016/j.brs.2017.01.578 28162976

[46] Huang H Jiang Y Xia M Tang Y Zhang T Cui H Increased resting-state global functional connectivity density of default mode network in schizophrenia subjects treated with electroconvulsive therapy. Schizophr Res (2018) 197:192–9. 10.1016/j.schres.2017.10.044 29117910

[47] Gan JL Duan HF Cheng ZX Yang JM Zhu XQ Gao CY Neuroprotective Effect of Modified Electroconvulsive Therapy for Schizophrenia: A Proton Magnetic Resonance Spectroscopy Study. J Nerv Ment Dis (2017) 205:480–6. 10.1097/NMD.0000000000000652 28141630

[48] Wolf RC Nolte HM Hirjak D Hofer S Seidl U Depping MS Structural network changes in patients with major depression and schizophrenia treated with electroconvulsive therapy. Eur Neuropsychopharmacol (2016) 26:1465–74. 10.1016/j.euroneuro.2016.06.008 27424799

[49] Cao B Cho RY Chen D Xiu M Wang L Soares JC Treatment response prediction and individualized identification of first-episode drug-naive schizophrenia using brain functional connectivity. Mol Psychiatry (2020) 25:906–13. 10.1038/s41380-018-0106-5 29921920

[50] Leucht S Cipriani A Spineli L Mavridis D Orey D Richter F Comparative efficacy and tolerability of 15 antipsychotic drugs in schizophrenia: a multiple-treatments meta-analysis. Lancet (2013) 382:951–62. 10.1016/S0140-6736(13)60733-3 23810019

