# Supplementary material for: Neuroanatomical Features That Predict Response to Electroconvulsive Therapy Combined With Antipsychotics in Schizophrenia: A Magnetic Resonance Imaging Study Using Radiomics Strategy
Source: Front Psychiatry. 2020 May 21;11:456. doi: 10.3389/fpsyt.2020.00456 (PMC7253706; doi:10.3389/fpsyt.2020.00456)
Supplement: Supplementary file 1 [file DataSheet_1.docx]

Definition of Treatment-Resistant Schizophrenia

In 2017, nine clinical with expertise in schizophrenia and treatment-resistant schizophrenia (TRS) participated in a closed meeting, at which published literature in key areas of TRS research was reviewed.^1^ The consensus from the roundtable meeting that focused on defining TRS is “The recommended definition of TRS is failure to respond on any 2 AP medications, each at an adequate dose (ie, equivalent to ≥ 600 mg of chlorpromazine/d) and treatment duration.” This recommendation is consistent with the consensus guideline on the minimum and optimum diagnosis and terminology created by TRRIP working group.^2^ The lack of consensus on clinically relevant criteria for defining TRS is obvious in earlier reports, i.e., TRRIP 2017, NICE 2014, WFSBP 2012, and APA 2004 (see Table 1 in Ref 1).^2-5^

Criteria Used for the Previous and Current Studies

Previous publications in this Journal used the criteria different from mentioned above. In the TRS study by Wimberley et al,^6^ they “defined TRS as first occurrence of either clozapine initiation or hospitalization due to schizophrenia during antipsychotic treatment within 18 months after at least 2 periods of different antipsychotic monotherapy lasting at least 6 weeks each.” This treatment-based proxy, used in their previously published study,^8^ built on five documents, including international and Danish treatment guidelines and the Kane criteria. Moreover, the modified Kane criteria^9^ was used the study by Mouchlianitis et al.^7^ “These require that patients had previously received at least 2 sequential trials of different antipsychotics, each of at least 6 weeks’ duration at a daily dose of at least 400 mg of chlorpromazine (CPZ) equivalents, but had shown inadequate response, including persistent psychotic symptoms.”

Because our study started in 2016, we used APA practice guidelines,^5^ i.e., “Treatment resistance is defined as little or no symptomatic response to multiple (at least two) antipsychotic trials of an adequate duration (at least 6 weeks) and dose (therapeutic range).”

Indications for Use of Electroconvulsive Therapy

In The Practice of Electroconvulsive Therapy: Recommendations for Treatment, Training, and Privileging: A Task Force Report of the American Psychiatric Association, 2nd ed,^10^ “ECT is most commonly considered in patients with schizophrenia only after unsuccessful treatment with antipsychotic medication.” In the WFSBP Guidelines and in the APA guidelines, ECT was recommended with limited evidence for the management of TRS.^4,5^ Furthermore, an acute episode and suicidal behavior/suicide attempts in schizophrenia are indications for use of ECT according to Chinese guideline and consensus.^11-13^

Taken together, in the current study, 33 patients were those with TRS, 20 patients were those during an acute episode, and 4 patients were those with suicide attempts (please see the supplementary table).

References:

1. Kane JM, Agid O, Baldwin ML, et al. Clinical Guidance on the Identification and Management of Treatment-Resistant Schizophrenia. *J Clin Psychiatry*. 2019;80:18com12123.

2. Howes OD, McCutcheon R, Agid O, et al. Treatment-Resistant Schizophrenia: Treatment Response and Resistance in Psychosis (TRRIP) Working Group Consensus Guidelines on Diagnosis and Terminology. *Am J Psychiatry*. 2017;174:216-229.

3. National Institute for Health and Care Excellence. Psychosis and schizophrenia in adults: prevention and management. https://www.nice.org.uk/guidance/cg178.

4. Hasan A, Falkai P, Wobrock T, et al. World Federation of Societies of Biological Psychiatry (WFSBP) Guidelines for Biological Treatment of Schizophrenia, part 1: update 2012 on the acute treatment of schizophrenia and the management of treatment resistance. *World J Biol Psychiatry*. 2012;13:318-378.

5. Lehman AF, Lieberman JA, Dixon LB, et al. Practice guideline for the treatment of patients with schizophrenia, second edition. *Am J Psychiatry*. 2004;161(2 Suppl):1-56.

6. Wimberley T, Gasse C, Meier SM, Agerbo E, MacCabe JH, Horsdal HT. Polygenic Risk Score for Schizophrenia and Treatment-Resistant Schizophrenia. *Schizophr Bull*. 2017;43:1064-1069.

7. Mouchlianitis E, Bloomfield MA, Law V, et al. Treatment-Resistant Schizophrenia Patients Show Elevated Anterior Cingulate Cortex Glutamate Compared to Treatment-Responsive. *Schizophr Bull*. 2016;42:744-752.

8. Wimberley T, Støvring H, Sørensen HJ, Horsdal HT, MacCabe JH, Gasse C. Predictors of treatment resistance in patients with schizophrenia: a population-based cohort study. *Lancet Psychiatry*. 2016;3:358-366.

9. Conley RR, Kelly DL. Management of treatment resistance in schizophrenia. *Biol Psychiatry*. 2001;50:898-911.

10. American Psychiatric Association. The Practice of Electroconvulsive Therapy: Recommendations for Treatment, Training, and Privileging: A Task Force Report of the American Psychiatric Association, 2nd ed. 2002.

11. Chinese Society of Psychiatry. Chinese Guidelines for Prevention and Treatment of Schizophrenia, 2nd ed. Beijing: Chinese Medical Multimedia Press; 2015.

12. Chinese Medical Doctor Association ECT and Neurostimulation Specialized Committee. Expert consensus on methods of electroconvulsive therapy (2017). *Clin Focus*. 2017;32:837-840. doi: 10.3969/j.issn.1004.

13. Chinese Association of Neurological Regulation Committee for Electroconvulsive Therapy and Nerve Stimulation, Chinese Association of Sleep Committee for Mental Psychology, Chinese Association of Anesthesiology. Expert consensus on modified electroconvulsive therapy (2019). *Transl Med J*. 2019;8(3):129-134.

Pattern classification analysis

A Leave-one-out cross-validation (LOOCV) framework was used to perform pattern classification analysis in the training set (Fig. 2) (Fan et al., 2011).^14^ In the LOOCV, one patient was used as a testing sample, and the remaining patients were applied as training samples to select features and build the classifier to classify the testing sample. Specifically, the best features based on univariate statistical tests (two-sample t-test) between responders and non-responders in the remaining patients were selected with P < .05 uncorrected, and then regularized multivariate logistic regression model (LRM) with the least absolute shrinkage and selection operator (LASSO) penalty was applied to select more important features and to build a classifier (Zhang et al., 2017).^15^ The regularization parameter λ that minimized the 10-fold cross-validation misclassification rate was selected, and then the model was constructed using training samples and the selected λ. By the way, most of the coefficients of the covariates were reduced to zero and the remaining non-zero coefficients were selected by LASSO. Finally, the constructed model was applied to classify the testing sample and was validated on the validation set. This procedure was repeated until each of patients in the training set had been used as the testing sample. Therefore, classification performance could be estimated based on all of the testing samples and be validated based on the averaged classification results of validation set. Moreover, the support vector machine (SVM) method was used to validate results with an independent classifier. Specifically, a SVM classifier with linear kernel was built upon the training samples using features selected by LASSO to classify the testing sample in the LOOCV. Ten-fold cross-validation was employed to determine the optimal value of the regularization parameter C. We tested values of C ∈ [0.01, 1] with a step size of 0.01 and then selected the C value that minimized the classification error in the remaining patients as the optimal regularization parameter. The SVM constructed using the optimal C value was applied to classify the testing sample and was validated on the validation set. As mentioned above, classification performance could be estimated based on all of the testing samples and be validated based on the averaged classification results of validation set.

Performance of the classifiers was measured quantitatively using the area under the receiver operating characteristic (ROC) curve (AUC), classification accuracy, sensitivity, specificity, positive predictive value, negative predictive value and phi correlation coefficient. AUC represents the classification power of a classifier, and a larger AUC indicates a better classification power. Phi correlation coefficient is a number between -1 and 1, with 1 indicating a perfect prediction, 0 indicating a random prediction, and values below 0 indicate a worse than random prediction (Table S4). The AUC was compared between training and validation cohorts using the DeLong test.

In order to obtain a model with fixed radiomic features and analyze the impact of clinical factors, we constructed the multivariate LRM based on the training set and validated it on the validation set. To obtain fixed radiomic features, we conducted the following analysis. Frequency of each feature selected by LASSO across 44 training partitions of the cross-validation setup was calculated to assess feature importance and to obtain a ranking of features (Table S6) (Dwyer et al., 2018).^16^ Features greater than 50% of the selected frequency were important and used as a feature set for further feature selection (Dwyer et al., 2018).^16^ The p value from the two-sample t-test of each feature and the Pearson correlation coefficient (r) between each pair of features were computed. All pairs of features with significant correlation were detected, and the feature in each of these pairs with the larger p value from the two-sample t-test was deleted from the feature set. The features of the deleted feature set as fixed radiomic features input into the radiomic LRM. To analyze the impact of clinical factors, negative score and antipsychotics dose combined with fixed radiomic features were also used to build the fusion LRM model. For these two LRM, we evaluated the multicollinearity according to the variance inflation factor (VIF) and tested the significance of regression coefficients (β) with t-tests for each independent variable (Kabacoff, 2015).^17^ If VIF was less than 4, there was no evidence of a multicollinearity problem (Kabacoff, 2015).^17^ If the β of an independent variable was not significant, it meant that this independent variable did not have a remarkable effect on the dependent variable, and could be deleted from the model. Performance of the LRM was measured quantitatively using AUC, classification accuracy, sensitivity, specificity, positive predictive value, negative predictive value and phi correlation coefficient in the training set and the validation set.

The following R packages were used for pattern classification analysis (http://www.R-project.org): The glmnet package was used for regularized multivariate logistic regression with LASSO. The e1071 package was used to perform the support vector machine. ROC curve is reported using the pROC package.

References:

14. Fan, Y., Liu, Y., Wu, H., Hao, Y., Liu, H., Liu, Z. & Jiang, T. (2011). Discriminant analysis of functional connectivity patterns on Grassmann manifold. Neuroimage 56, 2058-67.

15. Zhang, B., Tian, J., Dong, D., Gu, D., Dong, Y., Zhang, L., Lian, Z., Liu, J., Luo, X., Pei, S., Mo, X., Huang, W., Ouyang, F., Guo, B., Liang, L., Chen, W., Liang, C. & Zhang, S. (2017). Radiomics Features of Multiparametric MRI as Novel Prognostic Factors in Advanced Nasopharyngeal Carcinoma. Clin Cancer Res 23, 4259-4269.

16. Dwyer, D. B., Cabral, C., Kambeitz-Ilankovic, L., Sanfelici, R., Kambeitz, J., Calhoun, V., Falkai, P., Pantelis, C., Meisenzahl, E. & Koutsouleris, N. (2018). Brain Subtyping Enhances The Neuroanatomical Discrimination of Schizophrenia. Schizophr Bull 44, 1060-1069.

17. Kabacoff, R. I. (2015). R in Action: Data Analysis and Graphics with R Second Edition. Manning Publications.

Predictive performance

We calculated the frequency of each feature selected by LASSO across 44 training partitions of the cross-validation setup and obtained a ranking that characterized the importance of features (Table S6). The first 22 features with a selection frequency higher than 50% were selected as a feature set. 17 features were removed due to feature correlation and the remaining 5 features were used to train the LRM. One feature was removed due to the multicollinearity (VIF > 4), and another feature was removed because the regression coefficient was not significant. The final radiomic LRM included three features (2nd, 9th, 14th in the Table S6), and the detail of the model was shown in the Table S7. The LRM yielded an accuracy of 93.18% in the training set and yielded an accuracy of 84.62% in the validation set (Table S8).

**Table S1.** Detailed scanning parameters

|  | T1 |
| --- | --- |
| Repetition time (ms) | 8.2 |
| Echo time (ms) | 3.2 |
| Flip angle (°) | 12 |
| Field of view (mm^2^) | 256 × 256 |
| Matrix | 256 × 256 |
| Slice thickness (mm) | 1 |
| Section gap (mm) | 0 |
| Number of slices | 196 |

**Table S2.** Comparison of gray matter volume between responders and non-responders

| No. | AAL | Regions (> 50% volume) | Difference^a^ | Number of voxels |
| --- | --- | --- | --- | --- |
| 1 | 85 | Temporal_Mid_L | positive | 111 |
| 2 | 27 | Rectus_L | positive | 132 |
| 3 | 38 | Hippocampus_R | positive | 262 |
| 4 | 89 | Temporal_Inf_L | positive | 202 |
| 5 | 30 | Insula_R | positive | 371 |
| 6 | 14 | Frontal_Inf_Tri_R | positive | 167 |
| 7 | 29 | Insula_L | positive | 664 |
| 8 | 32 | Cingulum_Ant_R | positive | 135 |
| 9 | 14 | Frontal_Inf_Tri_R | positive | 123 |
| 10 | 46 | Cuneus_R | positive | 118 |
| 11 | 1 | Precentral_L | positive | 272 |
| 12 | 33 | Cingulum_Mid_L | positive | 138 |
| 13 | 34 | Cingulum_Mid_R | positive | 107 |
| 14 | 60 | Parietal_Sup_R | positive | 178 |
| 15 | 77 | Thalamus_L | negative | 212 |
| 16 | 63 | SupraMarginal_L | negative | 435 |
| 17 | 64 | SupraMarginal_R | negative | 112 |
| 18 | 49 | Occipital_Sup_L | negative | 133 |
| 19 | 62 | Parietal_Inf_R | negative | 163 |

^a^Positive, responders > non-responders; Negative, responders < non-responders.

**Table** S**3.** First-order statistics features

| No. | Name of feature | Abbreviation | Formula |
| --- | --- | --- | --- |
| 1 | sum | sum | $\sum_{i=1}^{N} X(i)$ |
| 2 | energy | energy | $\sum_{i=1}^{N} {X(i)}^{2}$ |
| 3 | kurtosis | kurtosis | $\frac{\frac{1}{N}\sum_{i}^{N} {(X\left( i \right)-\overline{X})}^{4}}{\left( \sqrt{\frac{\sum_{i}^{N} {(X\left( i \right)-\overline{X})}^{2}}{N}} \right)^{2}}$ |
| 4 | maximum | max | max($X(i)$) |
| 5 | mean | mean | $\frac{1}{N}\sum_{i=1}^{N} X(i)$ |
| 6 | mean absolute deviation | mad | $\frac{1}{N}\sum_{i=1}^{N} \left\vert X\left( i \right)-\overline{X} \right\vert$ |
| 7 | minimum | min | min($X(i)$) |
| 8 | median | mid | median($X(i)$) |
| 9 | range | range | max($X(i)$) - min($X(i)$) |
| 10 | root mean square | rms | $\sqrt{\frac{\sum_{i}^{N} {X(i)}^{2}}{N}}$ |
| 11 | skewness | skewness | $\frac{\frac{1}{N}\sum_{i}^{N} {(X\left( i \right)-\overline{X})}^{3}}{\left( \sqrt{\frac{\sum_{i}^{N} {(X\left( i \right)-\overline{X})}^{2}}{N}} \right)^{3}}$ |
| 12 | standard deviation | std | ${\frac{1}{N-1}\sum_{i=1}^{N} {(X\left( i \right)-\overline{X})}^{2}}^{1/2}$ |
| 13 | variance | var | $\frac{1}{N-1}\sum_{i=1}^{N} {(X\left( i \right)-\overline{X})}^{2}$ |
| 14 | entropy | entropy | $\sum_{i=1}^{N} P\left( i \right){log}_{2}P(i)$ |
| 15 | uniformity | uniformity | $\sum_{i}^{N} {P(i)}^{2}$ |

First-order statistics describe the distribution of voxel intensities within the image through commonly used and basic metrics. Let X denotes the three dimensional image matrix with N voxels and P the first order histogram with L discrete intensity levels. The formulas of 15 features are reference to Aerts HJ, Velazquez ER, Leijenaar RT, et al. Decoding tumour phenotype by noninvasive imaging using a quantitative radiomics approach. *Nat Commun* 2014;5:4006.

**Table** S**4.** Classification results of LRM and SVM based on LOOCV in the training set and the validation results in the validation set

| Metrics | | ACC | SENS | SPEC | PPV | NPV | AUC | PHI |
| --- | --- | --- | --- | --- | --- | --- | --- | --- |
| LRM | Training | 90.91% | 95.45% | 86.36% | 87.50% | 95.00% | 0.9318 | 0.8216 |
|  | Validation | 87.59% | 83.33% | 91.23% | 90.26% | 87.34% | 0.9031 | 0.7603 |
| SVM | Training | 90.91% | 100% | 81.82% | 84.62% | 100% | 0.9298 | 0.8321 |
|  | Validation | 91.78% | 85.61% | 97.08% | 97.02% | 89.29% | 0.9497 | 0.8447 |

ACC, accuracy; SENS, sensitivity; SPEC, specificity; PPV, positive predictive value; NPV, negative predictive value; AUC: area under the receiver operating characteristic curve; PHI, phi correlation coefficient.

**Table** S5**.** Frequency of features

| No. | No. 285 | No. ROI | Regions | Features | Count (44) | Frequency (i/44) |
| --- | --- | --- | --- | --- | --- | --- |
| 1 | 3 | 1 | Temporal_Mid_L | kurtosis | 44 | 1 |
| 2 | 112 | 8 | Cingulum_Ant_R | min | 44 | 1 |
| 3 | 104 | 7 | Insula_L | entropy | 43 | 0.977273 |
| 4 | 168 | 12 | Cingulum_Mid_L | kurtosis | 43 | 0.977273 |
| 5 | 246 | 17 | SupraMarginal_R | mad | 43 | 0.977273 |
| 6 | 44 | 3 | Hippocampus_R | entropy | 42 | 0.954545 |
| 7 | 53 | 4 | Temporal_Inf_L | mid | 42 | 0.954545 |
| 8 | 56 | 4 | Temporal_Inf_L | skewness | 42 | 0.954545 |
| 9 | 226 | 16 | SupraMarginal_L | sum | 42 | 0.954545 |
| 10 | 31 | 3 | Hippocampus_R | sum | 40 | 0.909091 |
| 11 | 183 | 13 | Cingulum_Mid_R | kurtosis | 40 | 0.909091 |
| 12 | 153 | 11 | Precentral_L | kurtosis | 37 | 0.840909 |
| 13 | 285 | 19 | Parietal_Inf_R | uniformity | 37 | 0.840909 |
| 14 | 35 | 3 | Hippocampus_R | mean | 35 | 0.795455 |
| 15 | 157 | 11 | Precentral_L | min | 35 | 0.795455 |
| 16 | 197 | 14 | Parietal_Sup_R | energy | 32 | 0.727273 |
| 17 | 125 | 9 | Frontal_Inf_Tri_R | mean | 31 | 0.704545 |
| 18 | 164 | 11 | Precentral_L | entropy | 28 | 0.636364 |
| 19 | 277 | 19 | Parietal_Inf_R | min | 26 | 0.590909 |
| 20 | 75 | 5 | Insula_R | uniformity | 25 | 0.568182 |
| 21 | 257 | 18 | Occipital_Sup_L | energy | 25 | 0.568182 |
| 22 | 196 | 14 | Parietal_Sup_R | sum | 24 | 0.545455 |
| 23 | 63 | 5 | Insula_R | kurtosis | 21 | 0.477273 |
| 24 | 1 | 1 | Temporal_Mid_L | sum | 18 | 0.409091 |
| 25 | 5 | 1 | Temporal_Mid_L | mean | 16 | 0.363636 |
| 26 | 138 | 10 | Cuneus_R | kurtosis | 15 | 0.340909 |
| 27 | 22 | 2 | Rectus_L | min | 13 | 0.295455 |
| 28 | 161 | 11 | Precentral_L | skewness | 13 | 0.295455 |
| 29 | 74 | 5 | Insula_R | entropy | 12 | 0.272727 |
| 30 | 119 | 8 | Cingulum_Ant_R | entropy | 11 | 0.25 |
| 31 | 200 | 14 | Parietal_Sup_R | mean | 9 | 0.204545 |
| 32 | 256 | 18 | Occipital_Sup_L | sum | 8 | 0.181818 |
| 33 | 233 | 16 | SupraMarginal_L | mid | 6 | 0.136364 |
| 34 | 7 | 1 | Temporal_Mid_L | min | 5 | 0.113636 |
| 35 | 34 | 3 | Hippocampus_R | max | 5 | 0.113636 |
| 36 | 227 | 16 | SupraMarginal_L | energy | 5 | 0.113636 |
| 37 | 45 | 3 | Hippocampus_R | uniformity | 4 | 0.090909 |
| 38 | 128 | 9 | Frontal_Inf_Tri_R | mid | 4 | 0.090909 |
| 39 | 262 | 18 | Occipital_Sup_L | min | 4 | 0.090909 |
| 40 | 195 | 13 | Cingulum_Mid_R | uniformity | 3 | 0.068182 |
| 41 | 18 | 2 | Rectus_L | kurtosis | 2 | 0.045455 |
| 42 | 94 | 7 | Insula_L | max | 2 | 0.045455 |
| 43 | 139 | 10 | Cuneus_R | max | 2 | 0.045455 |
| 44 | 140 | 10 | Cuneus_R | mean | 2 | 0.045455 |
| 45 | 158 | 11 | Precentral_L | mid | 2 | 0.045455 |
| 46 | 230 | 16 | SupraMarginal_L | mean | 2 | 0.045455 |
| 47 | 235 | 16 | SupraMarginal_L | rms | 2 | 0.045455 |
| 48 | 243 | 17 | SupraMarginal_R | kurtosis | 2 | 0.045455 |
| 49 | 48 | 4 | Temporal_Inf_L | kurtosis | 1 | 0.022727 |
| 50 | 121 | 9 | Frontal_Inf_Tri_R | sum | 1 | 0.022727 |
| 51 | 151 | 11 | Precentral_L | sum | 1 | 0.022727 |
| 52 | 156 | 11 | Precentral_L | mad | 1 | 0.022727 |
| 53 | 198 | 14 | Parietal_Sup_R | kurtosis | 1 | 0.022727 |
| 54 | 203 | 14 | Parietal_Sup_R | mid | 1 | 0.022727 |
| 55 | 228 | 16 | SupraMarginal_L | kurtosis | 1 | 0.022727 |
| 56 | 247 | 17 | SupraMarginal_R | min | 1 | 0.022727 |

**Table** S**6.** Classification results of reconstructed LRM and SVM after removal of one patient without receiving antipsychotics

| Metrics | | ACC | SENS | SPEC | PPV | NPV | AUC | PHI |
| --- | --- | --- | --- | --- | --- | --- | --- | --- |
| LRM | Training | 90.91% | 95.45% | 86.36% | 87.50% | 95.00% | 0.9318 | 0.8216 |
|  | Validation | 87.12% | 84.47% | 89.77% | 90.42% | 86.61% | 0.9034 | 0.7561 |
| SVM | Training | 90.91% | 100% | 81.82% | 84.62% | 100% | 0.9298 | 0.8321 |
|  | Validation | 90.91% | 85.61% | 96.21% | 96.59% | 87.58% | 0.9407 | 0.8298 |

ACC, accuracy; SENS, sensitivity; SPEC, specificity; PPV, positive predictive value; NPV, negative predictive value; AUC: area under the receiver operating characteristic curve; PHI, phi correlation coefficient.

**Table S7.** Variables and coefficients of the radiomic LRM

| Variable | Radiomic LRM | | |
| --- | --- | --- | --- |
|  | β | Adjusted OR (95% CI) | *P* value |
| Intercept | 0.059 |  | 0.9349 |
| Min of Cingulum_Ant_R | 2.270 | 9.680 (2.457-103.819) | 0.0110^*^ |
| Sum of SupraMarginal_L | -2.547 | 0.078 (0.003-0.407) | 0.0341^*^ |
| Mean of Hippocampus_R | 3.168 | 23.752 (3.728-500.296) | 0.0077^*^ |

β is the regression coefficient. ^*^*P* ＜ 0.05. OR, odds ratio; CI, confidence interval.

**Table S8.** Classification results of the radiomic LRM

| Metrics | ACC | SENS | SPEC | PPV | NPV | AUC | PHI |
| --- | --- | --- | --- | --- | --- | --- | --- |
| Training set | 93.18% | 95.45% | 90.91% | 91.30% | 95.24% | 0.9711 | 0.8645 |
| Validation set | 84.62% | 83.33% | 85.71% | 83.33% | 85.71% | 0.8571 | 0.6905 |

ACC, accuracy; SENS, sensitivity; SPEC, specificity; PPV, positive predictive value; NPV, negative predictive value; AUC: area under the receiver operating characteristic curve; PHI, phi correlation coefficient.

**
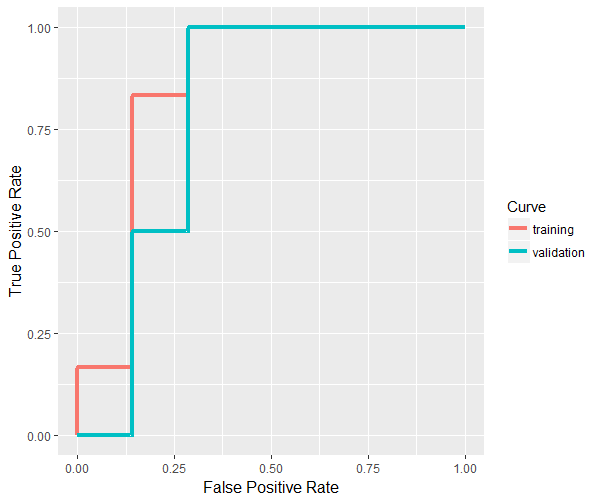
**

**Figure S1. Areas under ROC curves in the training set and validation set of the radiomic LRM.**

**
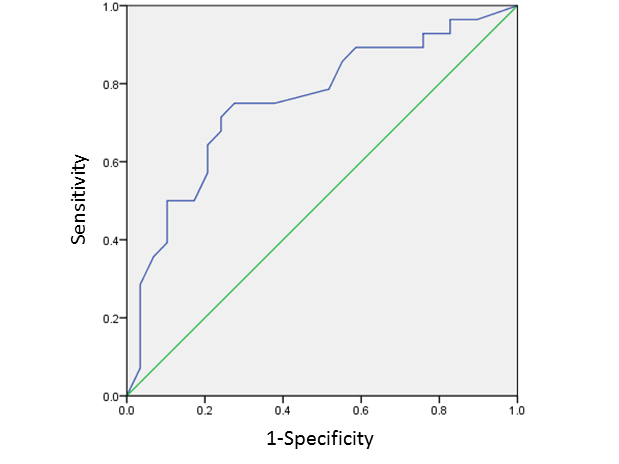
**

**Figure S2. Predictive function of PANSS negative score at baseline**

ROC analysis showed the area under curve for PANSS negative score at baseline was 0.754 (*P* = 0.001; 95% CI, 0.626, 0.883) to distinguish responders from non-responders. Notably, when PANSS negative score was set at 24.5, sensitivity, specificity, and accuracy was 75.0%, 72.4%, and 73.7%, respectively.
